# High yield production and purification of two recombinant thermostable phosphotriesterase-like lactonases from *Sulfolobus acidocaldarius* and *Sulfolobus solfataricus* useful as bioremediation tools and bioscavengers

**DOI:** 10.1186/s12896-018-0427-0

**Published:** 2018-03-20

**Authors:** Odile Francesca Restaino, Maria Giovanna Borzacchiello, Ilaria Scognamiglio, Luigi Fedele, Alberto Alfano, Elena Porzio, Giuseppe Manco, Mario De Rosa, Chiara Schiraldi

**Affiliations:** 1 Department of Experimental Medicine, Section of Biotechnology and Molecular Biology, University of Campania “Luigi Vanvitelli”-ex Second University of Naples, Naples, Italy; 2 Institute of Protein Biochemistry, National Research Council of Italy, Naples, Italy

**Keywords:** Archaea, Extremozymes, Fed-batch fermentation, Organophosphates, Thermostable phosphotriesterase-like lactonase, Ultra-filtration membrane-based purification

## Abstract

**Background:**

Thermostable phosphotriesterase-like lactonases (PLLs) are able to degrade organophosphates and could be potentially employed as bioremediation tools and bioscavengers. But nowadays their manufacturing in high yields is still an issue that limits their industrial applications. In this work we aimed to set up a high yield production and purification biotechnological process of two recombinant PLLs expressed in *E. coli*, the *wild type Sac*Pox from *Sulfolobus acidocaldarius* and a triple mutated *Sso*Pox C258L/I261F/W263A, originally from *Sulfolobus solfataricus.* To follow this aim new induction approaches were investigated to boost the enzyme production, high cell density fermentation strategies were set-up to reach higher and higher enzyme yields up to 22-L scale, a downstream train was studied to meet the requirements of an efficient industrial purification process.

**Results:**

Physiological studies in shake flasks demonstrated that the use of galactose as inducer increased the enzyme concentrations up to 4.5 folds, compared to the production obtained by induction with IPTG. Optimising high cell density fed-batch strategies the production and the productivity of both enzymes were further enhanced of 26 folds, up to 2300 U·L^− 1^ and 47.1 U·L^− 1^·h^− 1^ for *Sac*Pox and to 8700 U·L^− 1^ and 180.6 U·L^− 1^·h^− 1^ for *Sso*Pox C258L/I261F/W263A, and the fermentation processes resulted scalable from 2.5 to 22.0 L. After being produced and extracted from the cells, the enzymes were first purified by a thermo-precipitation step, whose conditions were optimised by response surface methodology. A following ultra-filtration process on 100 and 5 KDa cut-off membranes drove to a final pureness and a total recovery of both enzymes of 70.0 ± 2.0%, suitable for industrial applications.

**Conclusions:**

In this paper, for the first time, a high yield biotechnological manufacturing process of the recombinant enzymes *Sac*Pox and *Sso*Pox C258L/I261F/W263A was set-up. The enzyme production was boosted by combining a new galactose induction approach with high cell density fed-batch fermentation strategies. An efficient enzyme purification protocol was designed coupling a thermo-precipitation step with a following membrane-based ultra-filtration process.

**Electronic supplementary material:**

The online version of this article (10.1186/s12896-018-0427-0) contains supplementary material, which is available to authorized users.

## Background

**Fig. 1:**
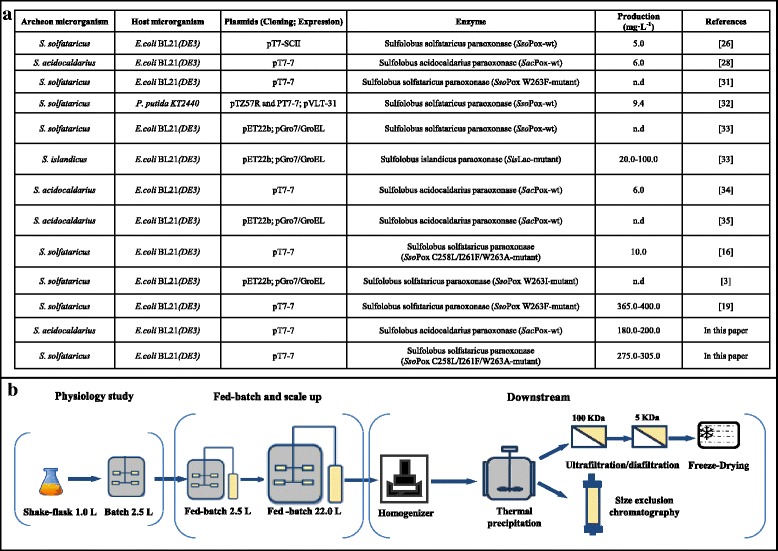
State of the art of the literature data on the isolation, expression, engineering and production of the thermostable phosphotriesterase-like lactonase enzymes with the relative references (**a**). Scheme of the different production and purification steps investigated in this paper to develop a complete biotechnological process for *Sac*Pox and *Sso*Pox 3 M manufacturing (**b**)

The thermostable phosphotriesterase-like lactonase (PLL) extremozymes from the archaeon strains of the *Sulfolobus* genera (mainly *Sulfolobus acidocaldarius*, *Sulfolobus islandicus*, *Sulfolobus solfataricus*) are able to hydrolyze the organophosphates (OPs), highly toxic compounds that irreversibly inhibit the acetylcholinesterase (AChE) and compromise the functionality of the nervous system of the target organisms (Fig. 1a) [1–4]. OPs like chlorpyrifos, parathion and paraoxon are massively employed as pesticides in intensive agriculture and nowadays they represent the major world environmental pollutants [4–6]. The release of OPs in the soil, in potable water sources and on edible cultures constitutes a risk for both animal and human health and the World Health Organization has estimated that OP ingestion causes every year more than 3 million people intoxications and about 250000 dead [7–10]. OPs like sarin or soman are also employed as chemical warfare nerve agents (CWNAs) and, although their use has been officially banned by the United Nations Organization, they have been recently employed in both war conflicts (e.g. in Syria in 2015–2017) and terroristic attacks (e.g. in Japan, in Matsumoto and Tokyo subway in 1994 and 1995) [11, 12]. PLL enzymes could be potentially employed as biocatalysists in OP degradation and removal in different fields: in environmental bioremediation as friendly and economic tools in alternative to chemical or physical decontamination approaches, in food safety and industry to remove pesticides from groceries and fruits, in public health and defense as valid exogenous bioscavengers in small defense systems and as innovative biomedical countermeasures for the quick sequester and inactivation of OPs in case of human exposure [1–3, 13–19]. Thanks to their extremely high thermal stability, their resistance to organic solvents, their ability to operate in wide pH ranges, in different buffers and in harsh conditions like in presence of surfactants and in outdoor, as well for the possibility of long-term storage at room temperatures, these extremozymes demonstrated to be more industrially attractive than their mesophilic counterparts, such as the phosphotriesterase enzymes (PTEs) from *Flavobacterium sp.* strains*, Brevundimonas diminuta, Pseudomonas diminuta* or *Agrobacterium radiobacter* P230 whose industrial applications has been limited so far by the low stability in solution and at temperature conditions higher than 30 °C [1, 20]. But to meet the industrial requirements and to be used in large scale decontamination processes these extremozymes have to show high catalytic efficiency, versatility as well as they have to be easily manufactured in high yields on large scales with limited costs. In the last decade emerging directed evolution strategies and molecular biology engineering tools have been employed to improve the catalytic activity and efficiency of these extremozymes up to several orders of magnitude, to modify their enantiomeric and stereo-selectivity and to express them in mesophilic strains, like in *E. coli* [2, 3, 16, 21–35] (Fig. 1a). These wild type and/or engineered extremozymes have been extensively characterized from a structural, biochemical and functional points of view but they have been produced so far only in few milligrams on liter concentrations, in the range from 5 to 10 mg·L^− 1^, or their production was so low that was not even reported [3, 16, 26, 28, 31–35] (Fig. 1a). Only in case of *Sis*Pox a concentration up to 100 mg·L^− 1^ was reached [33], but all the PLL manufacturing processes reported in literature have been lab-scale, low density shake flask or batch fermentations (Fig. 1a). A single mutant form of *Sso*Pox (*Sso*Pox W263F) [31] represents the only PLL enzyme produced at higher yields on pre-industrial vessels [19] (Fig. 1.a). Thus the design of large-scale, high yield biotechnological production and purification processes for most of these PLL enzymes is still an unresolved issue. In this experimental work we focused our attention on two enzymes, the *wild type Sac*Pox from *S. acidocaldarius* and a triple mutated form of *Sso*Pox (*Sso*Pox 3 M, with the mutated residues C258L/I261F/W263A) both previously isolated, in case of *Sso*Pox 3 M modified by applying in vitro protein evolution strategies, and expressed in soluble forms in *Escherichia coli* BL21. The two enzymes were structurally and biochemically characterized and their activity, kinetic constants and substrate affinity were determined [16, 28]. Both of them were active in a pH range from 5.0 to 9.0 (with a maximum from 8.0 to 8.5) and in a temperature range from 10 to 100 °C (with a maximum from 65 to 75 °C), but they showed different substrate specificity and specific activity; in fact the engineered *Sso*Pox 3 M hydrolysed the paraoxon with a specific activity 10 folds higher than *Sac*Pox (122.6 vs 12.03 U·mg ^− 1^), but *Sac*Pox was able to degrade better OPs like methyl-paraoxon, parathion and methyl-parathion [16, 34]. Furthermore both enzymes have already demonstrated, in small scale tests, to be employable in cleaning OPs from different surfaces like glass, tissues and fruits, also in presence of surfactants and even when dissolved in tap water [16]. So far these enzymes have been produced in 8-L shake flasks on LB medium from the engineered *E. coli* strains, by inducing with 1.0 mM IPTG at the beginning of the exponential growth phase, and their final concentrations reached values in the range from 6.0 to 10.0 mg·L^− 1^. They have been purified, after being extracted from the cells, by coupling a long three step thermal-precipitation procedure with two different chromatographic phases on affinity, size-exclusion and/or anion exchange columns (Fig. 1a) [16, 28]. Although these procedures proved to be satisfactory to obtain sufficient enzyme amounts to permit a structural characterization, they are not applicable in an industrial production perspective because of the low yields and the high costs, thus alternative strategies have to be found. IPTG, for example, is commonly used as inducer for the expression of recombinant proteins from *E. coli* strains in lab scale processes, but it generally slows down the growth and at concentrations higher than 2.0 mM it results even toxic for the bacterial cells and that limits its use as inducer in high cell density cultivations [36]. In recent recombinant protein production processes galactose have been employed in addition or in alternative to IPTG; it is not toxic for the cell growth and it is able to enhance the protein production of at least 10 folds [19, 37–39]. Fed-batch strategies could be employed in alternative to batch growths and could be designed in order to obtain an economical reliable and scalable fermentation process for enzyme production. In this perspective the bacterial nutritional requirements and metabolic outputs have to be supported by formulating appropriate feeding and aeration profiles to drive growth up to high cell density and to obtain enzyme production with high yields, trying to avoid any overflow metabolism and/or anaerobic conditions as well as any inhibiting by-product formation, like acetic acid [19, 39–41]. To achieve this aim the use of glycerol as carbon source in the fermentation medium, instead of glucose, is to be preferred because it is more slowly up-taken by the bacterial cells thus leading to a reduction in acetic acid formation. Also the modulation of the stirring and the airflow in the vessel during the growth are critical parameters to be taken in account [42–44]. A good biotechnological process should also include an efficient downstream process able to assure high recovery of the product with a suitable purity grade, at limited economic costs. In case of thermophilic recombinant enzymes the first step of the purification train, after the extraction from the bacterial cells, is generally a thermal-precipitation process that takes advantage of the thermal stability of the extremozymes compared to the mesophilic proteins. Different factors like the temperature, the number of steps of precipitation, the initial total protein concentration or the presence of cofactors could influence the process and have to be wisely investigated. Response surface methodology (RSM) is frequently used to set up parameters in bioprocess design and could be a useful tool to optimize the factors in a purification train [45, 46]. An ultra-filtration step could be included in a protein purification train in alternative to multiple chromatographic separation, because suitable for industrial applications, economic and easy to be scaled-up [47–49]. In the present research we attempted at producing and purifying both *Sac*Pox and *Sso*Pox 3 M in high yields up to pilot scale. For boosting the enzyme expression new induction conditions were investigated in shake flaks and in batch fermentations, while appropriate fed-batch strategies were applied to support both growth and enzyme yield. For designing an efficient purification train a RSM analysis was employed to better evaluate the thermal-precipitation parameters, while a following ultra-filtration protocol was assessed as a reliable downstream process towards enzyme scale up and manufacturing. (Fig. 1b).

## Methods

### Microorganism strains and chemicals

The strains *E. coli BL21* (DE3)-*Sacpox* and *E. coli BL21* (DE3)-*Ssopox* 3 M (C258L/I261F/W263A) were obtained by Prof. Manco by employing genetic engineering strategies, as previously described [16, 28], and they were stored at − 80 °C in 20% (*v*/v) glycerol stock solutions. The cells were propagated and grown on a semi-defined medium [20 g∙L^− 1^ glycerol, 2.4 g∙L^− 1^ yeast extract, 1.2 g∙L^− 1^ tryptone, 4.3 g∙L^− 1^ KH_2_PO_4_, 17.4 g∙L^− 1^ K_2_HPO_4,_ 1.0 g∙L^− 1^ (NH_4_)_2_SO_4_, 1.25 g∙L^− 1^ MgCl_2_, 3.0 g∙L^− 1^ citric acid, 0.3 g∙L^− 1^ thiamine, 1.25 g∙L^− 1^ MgCl_2_·6H_2_O, 12.9 mg∙L^− 1^ MnSO_4_·H_2_O and 10.0 ml∙L^− 1^ of a trace metal solution consisting of 0.02 g∙L^− 1^ FeSO_4_·3H_2_O, 0.02 g∙L^− 1^ CaCl_2_·H_2_O, 8.7 mg∙L^− 1^ ZnSO_4_·7H_2_O, 3.2 mg∙L^− 1^ CuCl_2_·2H_2_O, 6.45 mg∙L^− 1^ CoCl_2_·2H_2_O and 2.7 g∙L^− 1^ Na_2_MoO_4_·2H_2_O], after it was sterilized in autoclave [19]. All the media components, except for the yeast extract (Organo-Technie, France) and the tryptone (OXOID, UK), were from Sigma-Aldrich (Italy), as well as the IPTG, the galactose (Gal) and the ampicillin. The antibiotic (100.0 μg∙L^− 1^) and the inducers were added to the medium after sterilization by filtration through a 0.22 μm membranes (Millipore, France). All the standards and solvents used in the purification protocols, in the assays and in the analytical methods were from Sigma-Aldrich (Italy) too.

### Microorganism growth

#### Shake flask experiments

*E. coli-Sacpox* and *E. coli-Ssopox* 3 M shake flask experiments were run in triplicate in 1.0-L baffled shake flasks containing 0.2-L of the semi-defined medium, at 37 °C and 200 rpm in a rotary air shaker (Infors HT Incubator, Switzerland). Their inoculum was prepared seeding 100 μl of the cell stock solution in a 50 mL-tube containing 10 ml of the semi-defined medium and by growing it at 37 °C and 200 rpm in the shaker over night. During the runs 2–4 ml of culture were withdrawn every hour to evaluate the bacterial growth by measuring the absorbance of the samples at 600 nm (Spectrophotometer DU800, Beckman Coulter, USA). The growth rates (μ) between two different time points (t) were calculated according to the formula μ = (ln Abs_600nm2_-ln Abs_600nm1_)/(t_2_-t_1_), with t_2_ > t_1_; the μ values at the different time points of three identical replicates were then averaged. At around 1.0 Abs_6oonm_ the cultures were induced with 0.01 or 0.1 or 0.5 or 1.0 mM IPTG, or with 5.0 or with 10.0 mM galactose and with 0.5 mM MnCl_2_ for *E. coli-Sacpox* or 0.5 mM CoCl_2_ for *E. coli-Ssopox* 3 M. Experiments were stopped at 5 hours post-induction, the final biomasses were collected by centrifugation at 4 °C and 6000 rpm for 30 min (Avanti J-20XP, Beckman Coulter, USA), stored at − 20 °C and then extracted by sonication to determine the final total protein and the phosphotriesterase production.

#### Batch and fed-batch experiments

*E. coli-Sacpox* and *E. coli-Ssopox* 3 M fermentations were run in 2.5-L and 22.0-L vessels (Biostat CT and Biostat C, Braun Biotech International, Sartorius Group, Germany) that had working volumes of 2.0 L and 15.0 L, respectively; both of them were sterilizable in situ and equipped with pH, temperature and pO_2_ probes and four peristaltic pumps for the addition of alkali, acid, antifoam and, eventually, feeding solutions. The pO_2_ electrodes (Mettler Toledo, Switzerland) were calibrated using a pure oxygen flow as 100% value. During all the fermentations the pH value was kept constant at 7.0 with the addition of 30% (*v*/v) NH_4_OH and/or 30% (v/v) H_2_SO_4_ solutions, the temperature was kept at 37 °C and the pO_2_ value inside the vessel was always kept higher than 20% by modulating the stirring in the range from 400 to 600 rpm and the airflow value between 0.75 and 1.0 vvm or, ultimately, by insufflating a pure oxygen flow. These process parameters were remotely controlled and collected by a Digital Control Unit (DCU) using a MFCS-win software (Braun Biotech International, Sartorius Group, Germany). Over-night grown shake flask cultures were used as inoculum for both batches and fed-batches (the inoculum volume was 2–3% of the medium volume). Batch experiments (2.5 L) were run for 24 h inducing at 6.0 Abs_6oonm_, at around the 6th hours of growth, with 1.0 mM IPTG, or 5.0 or 10.0 mM galactose plus 0.5 mM MnCl_2_ for *E. coli-Sacpox* or 0.5 mM CoCl_2_ for *E. coli-Ssopox* 3 M. In fed-batch experiments (2.5 and 22.0 L), after 8–9 h of batch phase, the cultures were fed with a concentrated nutrient solution (160.0 g∙L^− 1^ of glycerol, 19.2 g∙L^− 1^ of yeast extract and 9.6 g∙L^− 1^ tryptone) following an exponential feeding profile (1.5 g∙L^− 1^∙h ^− 1^ of glycerol from 8 to 14 h of growth, 2.0 g∙L^− 1^∙h^− 1^ from 14 to 20 h and 2.5 g∙L^− 1^∙h^− 1^ from 20 h up to the end). Induction was performed at around 24 h of growth, between 38 and 42 Abs_6oonm_, with 5.0 mM or 10.0 mM galactose plus 0.5 mM MnCl_2_ or 0.5 mM CoCl_2_ and experiments were stopped at 24 h after induction. Broth samples were withdrawn at regular times in both batches and fed-batches to determine the bacterial growth by absorbance, as described above, or by weighting the wet biomass of 3 ml broth samples (cell wet weight per volume-g_cww_∙L^− 1^), obtained after centrifugation at 4 °C and 6000 rpm for 10 min (Avanti J-20XP, Beckman Coulter, USA). The supernatants of these broth samples were used for determining the concentration of glycerol, acetic acid and of inducers at the different time points of the growth. For determining also the kinetic of the total protein and of phosphotriesterase production, samples of the cultures were withdrawn before and after induction and the biomasses were collected by centrifugation as reported above. The batch and fed-batch final fermentation broths were harvested and centrifuged at 4 °C and 6000 rpm for 40 min (Avanti J-20XP, Beckman Coulter, USA) and the recovered biomasses stored at − 20 °C.

### Phosphotriesterase purification process

#### Enzyme extraction and thermal precipitation

The final biomasses of shake flask experiments or the pellets of the different time points of the fermentation runs were re-suspended in a lysis buffer (20 mM hepes, 0.5 mM MnCl_2_ for *Sac*Pox and 0.5 mM CoCl_2_ for *Sso*Pox 3 M or, 0.1% (*w*/*v*) Triton X at pH 8.5) in a ratio of 1 to 3 (w/v) and disrupted by sonication (Sonicator 3000, Misomix, USA) (10 min, 30 s on and 30 s off; power level of 3.5) to extract the enzymes, as previously described [19]. The final fed-batch fermentation biomasses (600 g_cww_) were re-suspended in the same lysis buffer and they were broken by a high pressure cell homogenizer (Emulsiflex C3, Avestin, Germany) applying a pressure of 15.000–20.000 psi (100.000–150.000 KPa). In all cases the cell debris were centrifuged at 4 °C and at 6500 rpm for 50 min (Avanti J-20XP, Beckman Coulter, USA), the crude extracts were recovered and a protease inhibitor solution was added to them (100 μl∙L^− 1^ as final concentration). Investigation and optimization of the parameters affecting the purification of the two enzymes by thermal precipitation was done by response surface methodology as described in the following paragraphs. Diverse small scale tests were performed to optimize the thermal precipitation: 2 ml-samples of crude extract, having an initial total protein concentration in a range from 48 to 0.48 g∙L^− 1^, were thermal precipitated at three different temperatures (60, 70, 80 °C) for 25 min, under a stirring range between 500 and 1200 rpm. Once optimized the conditions, small scale thermal precipitation of the crude extracts were performed at 70 °C and at 900 rpm for 25 min by using a thermomixer (Eppendorf, Germany), after having diluted the samples with the buffer solution in order to have a total protein concentration between 4.0 and 5.0 g∙L^− 1^, while large scale thermo-precipitations were performed in a 20-L glass jacked bio-reactor (Steroglass, Italy), equipped with a Rushton pale stirring system, at the same conditions. After the thermal precipitation step, in both small or large scale processes, the precipitated proteins were separated from the solutions by centrifugation at 6500 rpm and 4 °C for 50 min. In case of large scale purification the supernatants were then further purified on 100 and 5 kDa cut-off membranes or by chromatography. All the crude extracts and thermo-precipitated samples were assayed to determine the total protein concentration and the phosphotriesterase activity.

#### Ultra-filtration and gel-filtration chromatography

In order to purify on large scale the *Sac*Pox and *Sso*Pox 3 M enzymes after the thermal precipitation step, ultra-filtration on membranes was performed by using an automatic tangential flow filtration system (Uniflux 10, UNICORN, GE Healthcare, USA) connected to a software to control and to monitor the process parameters (Schiraldi et al. 2012). Because the two enzymes have a molecular weight of about 35.0 KDa [16, 28], the supernatants of the thermal precipitation (ranging from 8 to 13 L) were first treated on 100 KDa cut-off membranes, having a total filtering area of 0.1 m^2^ (Sartorius Group, Germany). After that 95% of the sample was filtered, a continuous diafiltration procedure was performed by adding to the retentate 5 volumes of a buffer solution (respect to the concentrated volume) containing 20.0 mM hepes and 0.2 mM CoCl_2_ or MnCl_2_, according to the phosphotriesterase. Both enzymes were present in the permeate that was then further concentrated on 5 kDa cut-off membranes (0.2m^2^ of total filtering area, Sartorius Group, Germany) and diafiltered with 2 volumes of buffer (respect to the concentrated volume). During the filtration process the trans membrane pressure (TMP) was calculated following the formula TMP = [(inlet pressure-retentate pressure)/2], considering that the out pressure was zero. Fluxes were calculated as volumes passed on a filtering area in an hour (LMH). Samples were taken during each ultra-filtration steps in order to determine the total protein concentration and the enzyme activity. At the end of the membrane processes the collected retentates were freeze-dried (Epsilon 2–6 D, Christ, Germany; method: 18 h at − 20 °C and at 1.05 mbar and then 3 h at 20 °C and at 0.040 mbar) and then stored at room temperature for 12 months; the activity of these samples was also checked by assays at different times of storage. Samples (5 ml) of supernatants from the large scale thermal precipitation processes of *Sac*Pox or *Sso*Pox 3 M were loaded on a gel-filtration column (Hiload 26/600, Superdex 75 pg, GE Healthcare, Italy) connected to a chromatographic system (ÄKTA explorer 100, GE Healthcare, Italy), equipped with two piston pumps, an UV detector, a pH meter, a conductivity cell and a fraction collector (Frac-950, GE Healthcare, Italy) and connected to a software to acquire the process data (Unicorn 5.0, GE Healthcare, Italy). Samples were loaded and eluted with a buffer (20.0 mM hepes, 0.5 mM MnCl_2_ for *Sac*Pox or 0.5 mM CoCl_2_ for *Sso*Pox 3 M, 0.1% (*w*/*v*) TritonX at pH 8.5) in isocratic conditions at a flow rate of 2.0 mL·min^− 1^ for 1.5 column volumes. Elution was monitored by contemporary detecting the absorbance at 280 nm; 2 mL-fractions were collected, the different peaks were pooled together and assayed to determine the total protein concentration and to test the phosphotriesterase activity.

### Analytical methods

#### High performance anion exchange and liquid chromatography

The supernatants of shake flask and fermentation broth samples (1 ml) were ultra-filtered on 10 kDa centrifugal filter devices (Amicon, USA) at 4 °C and 11000 rpm for 12 min (Centrifuge Z216 MK, Hermle Labortechnik GmbH, Germany). The filtered volumes were analysed by high performance anion exchange chromatography (HPAE-PAD) (ICS-3000, Dionex, USA) to determine the concentrations of residual glycerol and inducer in the medium and by HPLC (Ultimate 3000, Dionex, USA) to determine the organic acid production during the bacterial growth, according to methods previously described [19, 39, 41].

#### Total protein and phosphotriesterase activity determination

Samples of crude extracts from shake flask or fermentation experiments or samples from the different steps of the purification process were assayed to determine the total protein content by using a spectroscopy method [50] and the bovine serum albumin (BSA) as standard (Biorad, USA). The phosphotriesterase activity of *Sac*Pox and *Sso*Pox 3 M was determined by measuring the increase of absorbance at 405 nm due to the enzymatic hydrolysis performed at 25 or 70 °C of the paraoxon (di-ethyl-p-NP-phosphate), used as substrate, in *p*-nitro-phenol. 20 μg of each different enzyme were added to 1 ml of 20.0 mM hepes buffer, at pH 8.5, containing 0.5 mM of paraoxon and the absorbance increase at 405 nm (Spectrophotometer DU800, Beckman Coulter, USA) was measured in 2 min run as previously described [16, 28, 31]. One enzyme unit was defined as the amount of enzyme necessary to transform 1 μmole of paraoxon into *p*-nitro-phenol in one minute (*ε* = 21 mM·Abs^− 1^). The concentration of the enzyme in terms of U·L^− 1^ was calculated from the absorbance determined by the assay; the enzyme yield, as unit on cell wet weight (U·g_cww_^− 1^), was calculated by dividing the U·L^− 1^ concentration for the biomass concentration (g_cww_·L^− 1^), while the productivity, in terms of U·L^− 1^·h^− 1^, was calculated by diving the concentration for the hours of run. Instead the enzyme concentrations, in terms of mg·L^− 1^, were determined by dividing the U·L^− 1^ values for the specific activity of the pure enzyme (12.03 U·mg^− 1^ for *Sac*Pox and 123.6 U·mg^− 1^ for *Sso*Pox 3 M) [16, 34].

#### SDS-PAGE and western blot analysis

The change in the protein pattern at the different steps of the downstream process was analyzed by SDS-PAGE by loading 20 μg of total protein content for each sample on a 12.5% polyacrylamide and bis-acrylamide gel and then running the gel as already described [51]. A protein ladder of molecular weights in the range from 7 to 198 kDa (Prestained Standard, Invitrogen, USA) was used as ladder, while the gels were stained with coomassie (Coomassie Brilliant Blue R–250, BIORAD, USA) for 7–10 min and then de-stained for 4–8 h with a 10% (*v*/v) acetic acid and 10% (v/v) methanol solution. A densitometry system (GEL DOCTM EZ System, BIORAD, USA), coupled with the software IMAGE LABTM (BIORAD, USA), was used to acquire the gels in order to determine the relative percentages of the *Sac*Pox and *Sso*Pox 3 M enzymes compared to the total protein content. The two purified enzymes, obtained at the end of the purification process, were analyzed by Western blot analyses, by using a polyclonal rabbit antibody against *Sso*Pox wt, obtained in Prof. Manco’s lab, that is able to recognize both *Sac*Pox and *Sso*Pox 3 M enzymes. After the SDS-PAGE run, 100 ng of total proteins were transferred on PVDF sheets at 50 V and at 4 °C for 1.5 h. Wheets were treated with the blocking solution (1× PBS, 0.1% tween, 5% milk) for 1.5 h and then incubated in the same solution with primary rabbit polyclonal antibody for 2 h at room temperature. After several washes with a PBS solution containing 0.1% tween, the antibody binding was detected by using horseradish peroxidase (HRP)-conjugated secondary anti-rabbit antibody after 1 h incubation at room temperature and by using a chemiluminescence kit (kit ECL Western Blotting Substrate, Abcam, United Kindom) detecting immunopositive signals on X-ray films.

### Data and statistical analyses

A Box-Benhken design (BBD) was performed to optimize the recovery of both extremozymes during the thermal precipitation step by using three independent variables (total protein concentration = A, stirring = B and temperature = C) at three different levels (− 1; 0; 1) (corresponding to 0.48–4.8-48.0 g·L^− 1^ for the total protein concentration, 500–900-1200 rpm for the stirring; 60–70-80 °C for the temperature; respectively). The experimental results of the response surface design methodology were analyzed using the software Minitab 17 (GMSL s.r.l., UK) and resulted of 15 experimental points for each enzyme, performed in random order as described in Additional file 1: Table S1. All the results of both enzyme production and purification processes that are reported in the text, in tables and in figures are averages values of three independent experiments calculated with their standard deviations by a Microsoft Office Excel 2007 program (Microsoft, USA). All the calculated values, like the yield and the productivity, were averaged values as well. Statistical comparison between group of data, as for example between the results of the different shake flask runs, were determined by the t-student test and the data were considered significantly different if *p* values were lower than 0.05.

## Results

### Shake flask experiments

**Fig. 2:**
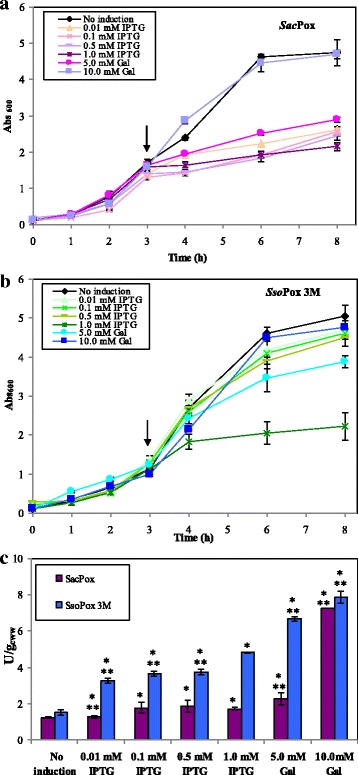
Shake flask growth curves of *E. coli-Sacpox* (**a**) and *E. coli-Ssopox* 3 M (**b**) induced with 0.01, 0.1, 0.5, 1.0 mM IPTG, 5.0 mM or 10.0 mM galactose at about 1.0 Abs_600nm_, as indicated by the arrows, compared with a not-induced growth; comparison of *Sac*Pox and *Sso*Pox 3 M enzyme production (U·g_cww_^−1^) in the different shake flask experiments (**c**). [*p < 0.05 compared to the not- induced shake flask; **p < 0.05 compared to the IPTG induced shake flask]

First physiological experiments were run in shake flasks to study the kinetic of growth of the two recombinant strains and to investigate the best induction conditions by using different concentrations of IPTG (in the range from 0.01 to 1.0 mM) or of galactose (at 5.0 and 10.0 mM), used as replacement of IPTG (Fig. 2a-c). In the not-induced experiments of both strains the final biomasses reached similar values (between 4.7 e 5.0 ± 0.1 Abs_600nm,_ respectively for *E. coli Sacpox* and *E. coli Ssopox* 3 M). The addition of IPTG, at any concentrations, caused a growth inhibiting effect on *E. coli Sacpox*, already after the first hour post induction, at this time the growth rates diminished in the range from 89 to 91% compared to the not induced runs (e.g. at one hour post induction the μ in the not induced shake flasks was 0.33 ± 0.02 h^− 1^ while in 1.0 mM IPTG runs the μ was 0.03 ± 0.01 h^− 1^). That drove to final biomass values 55.0% times lower than the not-induced experiments (Fig. 2a). In *E. coli Ssopox* 3 M experiments, instead, the growth rates, in the first hour post-induction, diminished less than in *E. coli Sacpox*, independently from the concentration of IPTG used, with values only 27%–30% lower than the control (e.g. at one hour post induction the μ was 0.87 ± 0.02 h^− 1^ in the not induced run and 0.60 ± 0.01 h^− 1^ in 1.0 mM IPTG experiments) (Fig. 2b). But in the following hours a higher inhibiting effect was noted for 1.0 mM IPTG induced runs, than for the other IPTG concentrations; that effect drove to a final biomass value 56.0% lower than the not induced run, similarly to what observed for *E. coli Sacpox* (Fig. 2b). The 5.0 mM Gal induction, instead, resulted in less inhibiting the growth rates of both strains, compared to IPTG (Fig. 2a-b); at one hour post induction the μ values were reduced of 48.0% and 22.0% respectively for *E. coli-Sacpox* and *E. coli-Ssopox* 3 M (μ values of 0.17 ± 0.02 h^− 1^ and 0.67 ± 0.02 h^− 1^, correspondently), while the final biomasses were diminished in the range from 30.0 to 38.0 ± 1.0%, compared to the not induced experiments (Fig. 2a-b). The induction with 10.0 mM galactose, instead, did not cause any significant reductions of the growth rates as well as of the final biomass values in both strains, compared to the not-induced experiments (differences were lower than 5%) (Fig. 2a-b). The phosphotriesterase production changed according to the type and the concentration of the inducer, as well as according to the strain (Fig. 2c). The two strains produced a similar basal enzyme concentration in the not-induced shake flask experiments (1.24 ± 0.03 U·g_cww_^− 1^ of *Sac*Pox and 1.51 ± 0.1 U·g_cww_^− 1^ of *Sso*Pox 3 M, equivalent to 15.61 ± 0.42 and 19.71 ± 3.8 U·L^− 1^, correspondently); but the addition of the inducer, independently from the type, drove always to a more consistent increase of *Sso*Pox 3 M than *Sac*Pox. In fact, compared to the not-induced experiments, the induction with IPTG, in the range from 0.10 to 1.0 mM, caused a 40.0% *Sac*Pox enhancement (in the range from 1.77 to 1.84 ± 0.05 U·g_cww_^− 1^) (no *Sac*Pox production increase were noted with 0.01 mM IPTG induction); while a 59.0% and a 68.0% improvement of *Sso*Pox 3 M (up to 4.82 ± 0.01 U·g_cww_^− 1^) were noted inducing with a concentration of IPTG in the range from 0.01 to 0.5 mM, or with 1.0 mM IPTG, respectively (Fig. 2c). The 5.0 mM galactose addition stimulated a higher enzyme production but, again, in different ways, up to 41.7% for *Sac*Pox (2.28 ± 0.32 U·g_cww_^− 1^) versus 77.3% for *Sso*Pox 3 M (6.66 ± 0.12 U·g_cww_^− 1^). The best results for both strains were reached by using 10.0 mM galactose that drove to a significant 66.8% increase of *Sac*Pox production (7.25 ± 0.03 U·g_cww_^− 1^, equivalent to 79.80 ± 0.03 U·L^− 1^) and to a 80.7% improvement of *Sso*Pox 3 M (7.86 ± 0.03 U·g_cww_^− 1^, equivalent to 87.00 ± 4.2 U·L^− 1^), respectively, values 5.0 times higher than the not-induced experiments (Fig. 2c). Using higher galactose concentrations no further improvement in enzyme production was noted and by employing 15.0 mM galactose an increase of 70.0% of *Sac*Pox and 82.0% of *Sso*Pox production was determined. Considering these data, we performed batch experiments to test 1.0 mM IPTG, 5.0 and 10.0 mM Gal induction conditions also in a controlled system, as in a fermentation vessel.

### Batch experiments

**Fig. 3:**
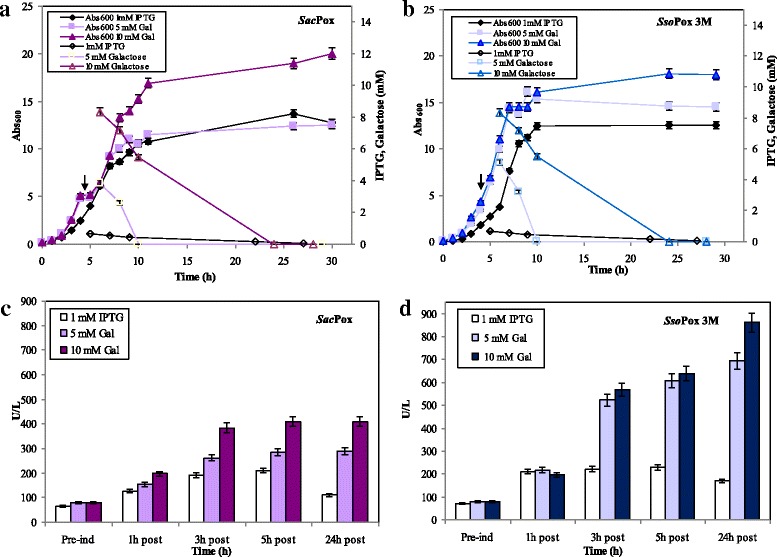
Batch experiments (2.5 L) of *E. coli-Sacpox* and *E. coli-Ssopox* 3 M induced with 1.0 mM IPTG, 5.0 or 10.0 mM galactose at around 6.0 Abs_600nm_, as indicated by the arrows: growth curves, IPTG or galactose up-take (**a**-**b**); *Sac*Pox and *Sso*Pox 3 M enzyme production (U·L^− 1^) in the different batch experiments (**c**-**d**)

Batch fermentations (2.5 L) were performed inducing with 1.0 mM IPTG, 5.0 or 10.0 mM galactose during the exponential phase at biomass values higher than in shake flask experiments (at 6.0 Abs_600nm_). As previously noted, the *E. coli Sacpox* growth curves and the final biomass values in batches induced with 1.0 mM IPTG or 5.0 mM galactose were similar (up to 12.6 ± 0.1 Abs_600nm_), differently from *E. coli Ssopox* 3 M experiments, where the final biomass in 5.0 mM galactose induced batch was 14% higher than the one obtained with IPTG induction (Fig. 3a-b). Higher final biomass values were obtained in case of 10.0 mM induction for both strains, up to 20.0 and 18.0 ± 2.0 Abs_600nm_ in *E. coli Sacpox* and *Ssopox* 3 M batches, correspondently. Maximum enzyme production in both strain batches were reached inducing with 10.0 mM galactose, but at different time points. *Sac*Pox reached its maximum at 5 h post induction with a production of 410.0 U·L^− 1^ and a productivity of 37.0 U·L^− 1^·h^− 1^ (values 2.0 and 1.5 folds higher than the IPTG and 5.0 mM galactose induced batch experiments and 5.0 times higher than the result obtained in shake flasks) (Fig. 3c). Instead *Sso*Pox 3 M maximum enzyme production was determined at 24 h post induction with a production of 867.5 U·L^− 1^ and productivity of 36.1 U·L^− 1^·h^− 1^ (a value 5.1 folds and 1.24 higher than the IPTG and 5.0 mM galactose induced batch experiments and almost 9.0 times higher than the result obtained in shake flasks) (Fig. 3d). Also in batch experiments the best results, in terms of growth and enzyme production, were obtained by inducing both strains with 10.0 mM galactose, and thus we decided to use this inducer at this concentration as the best one to be used in fed-batches experiments.

### Fed-batch experiments

**Fig. 4:**
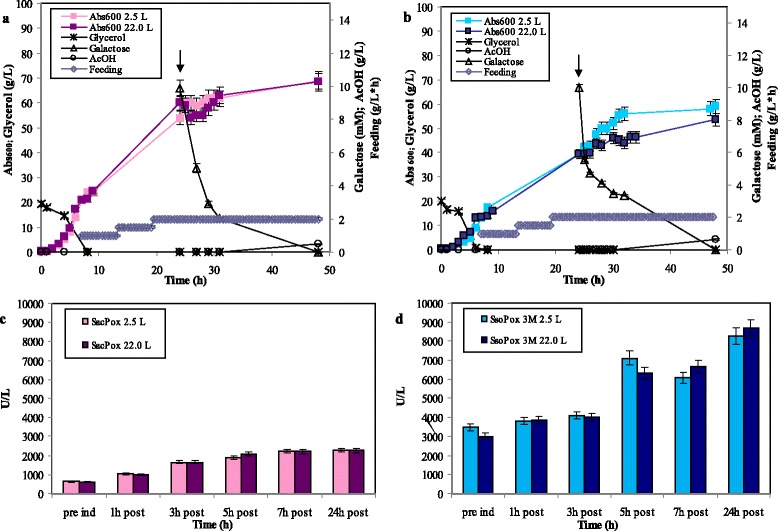
Fed-batch experiments in 2.5 and 22.0-L vessels of *E. coli Sacpox* and *E. coli Ssopox* 3 M induced with 10.0 mM galactose at about 40.0 Abs_600nm_, as indicated by the arrows: growth curves, galactose up-take, glycerol consumption, acetic acid formation and feeding profile (**a**-**b**). *Sac*Pox and *Sso*Pox 3 M enzyme production (U·L^− 1^) in the 2.5 and 22.0-L fed-batch experiments (**c**-**d**)

In fed-batch experiments (in 2.5 and 22.0-L vessels) a feeding profile was supplied to the two cultures at the end of the batch phase, that in absence of induction was around the 7-8th hours of growth (Fig. 4a-b). The growth of both strains was prolonged without accumulation of the carbon source, perfectly controlling the acetate formation and it resulted similar in both scales, up to a final biomass of 68.0 and 60.0 ± 2.0 Abs_600nm_ for *E. coli Sacpox* and *Ssopox* 3 M, correspondently; these final biomass values were about 3.0 times higher than in batch experiments (as reported before in batch experiments, also in fed-batches the *E. coli Sacpox* growth resulted slightly higher than the *E. coli Ssopox* 3 M growth) (Fig. 4a-b). The cultures were induced with 10.0 mM galactose at 24 h of growth, at higher biomass values compared to the batches experiments (at around 40.0 ± 3.0 Abs_600nm_), and that determined a higher enzyme production up to a maximum of about 2200.0 U·L^− 1^ (equivalent to 200.0 mg·L^− 1^) of *Sac*Pox at the 7th hour post induction and of about 8673.0 U·L^− 1^ (equivalent to 305.0 mg·L^− 1^) of *Sso*Pox 3 M at the 24th hour post induction (Fig. 4a-b). The enzyme production was similar in both scales for both strains and it resulted increased of 5.5 and 11.3 folds for *Sac*Pox and *Sso*Pox 3 M, correspondently, compared to the batches ones (Fig. 4c-d).

### Enzyme extraction and thermal precipitation

**Fig. 5:**
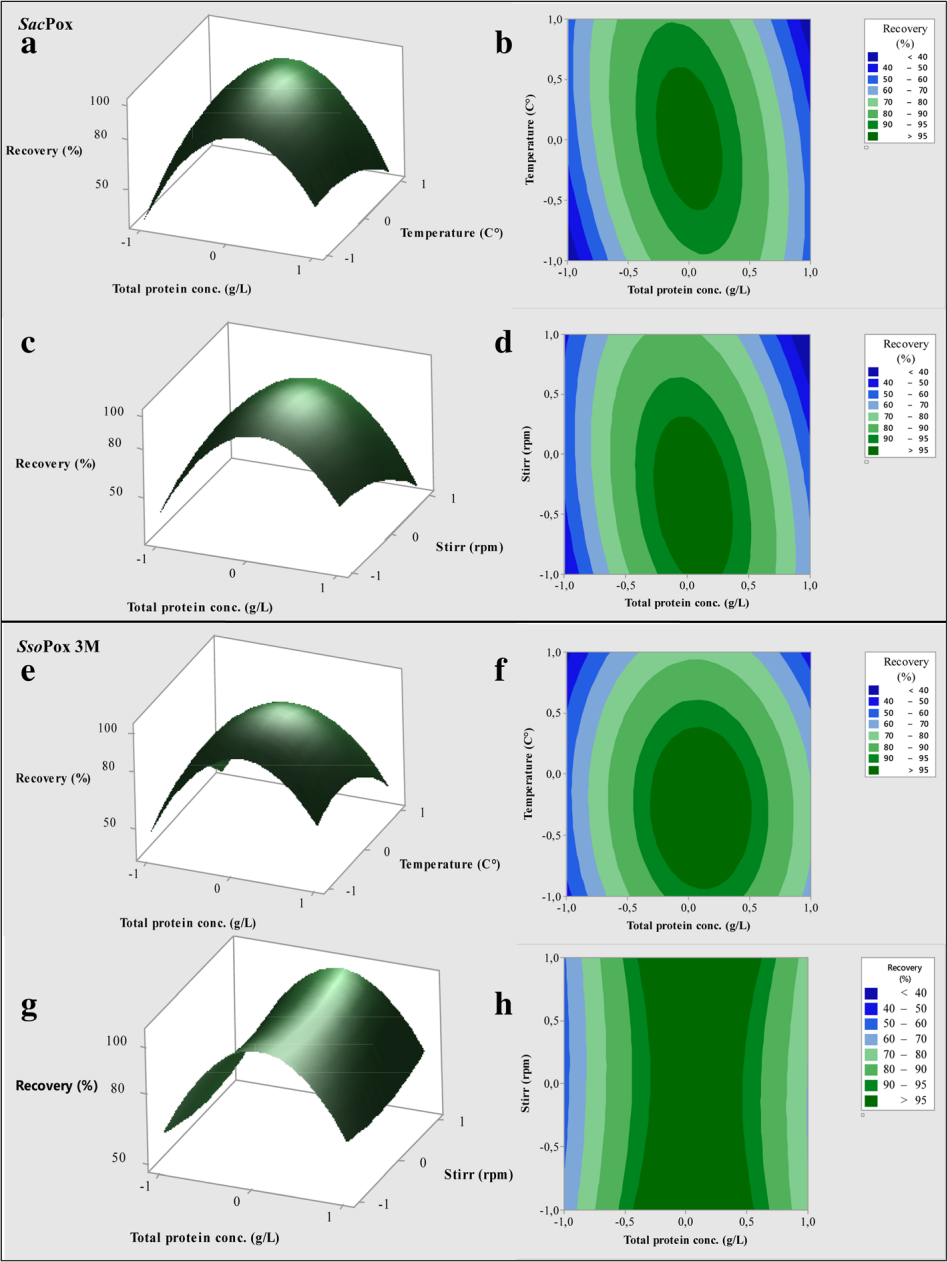
Response surface methodology (3D) showing the interactive effects of temperature, total protein concentration and stirring on the recovery of *Sac*Pox (**a**-**d**) and *Sso*Pox 3 M (**e**-**h**) enzymes in the process of thermal precipitation

*Sac*Pox and *Sso*Pox 3 M purification processes were designed considering that they are intracellularly expressed and thermostable enzymes (Fig. 1b; Table 1a-b). 600 g_cww_ of the wet final biomasses, obtained in the 22-L fed-batches, were first mechanically disrupted by an homogenizer: in the crude extracts a total of 12748 ± 380 *Sac*Pox enzyme units and of 58355 ± 1600 *Sso*Pox 3 M units were obtained, respectively (Table 1a-b). To optimize the parameters of the following thermal precipitation step, a response surface methodology was employed and small scale tests were performed (Fig. 5a-h; Additional file 1: Table S1). Results showed that both the initial total protein concentration of the crude extracts and the temperature used to perform the thermal precipitation influenced the process, but the initial total protein concentration resulted more critical for the thermal precipitation of *Sac*Pox while the temperature values resulted stricter for obtaining an optimal thermal precipitation of *Sso*Pox 3 M. In fact a *Sac*Pox enzyme recovery higher than 95% was obtained only when an initial total protein concentration of around 4.8 g·L^− 1^ ± 0.5 was used and temperature values in the range from 65.0 to 75.0 ± 0.5 °C, while the same recovery yield was obtained in the thermal precipitation of *Sso*Pox 3 M starting with a slightly wider initial total protein concentration range (3.7–5.9 ± 0.2 g·L^− 1^) in a stricter temperature range from 66.0 to 72.0 ± 0.5 °C (Fig. 5a-b and e-f). The stirring seemed to have a significant influence for the *Sac*Pox thermal precipitation process and values from 500 to 900 rpm drove to an enzyme recovery values higher than 96%, starting with an initial total protein concentration of 4.8 ± 0.5 g·L^− 1^. Differently the stirring values seemed not to influence the recovery yield in *Sso*Pox 3 M thermal precipitation process if the initial total protein concentration was around 4.8 ± 0.5 g·L^− 1^ (Fig. 5c-d and g-h). The fitted model was also analyzed by ANOVA; the low probability *p* values of the F values showed that the model had high significance and suitability as confirmed also by the adjusted determination coefficients (adjusted R^2^) (Additional file 2: Table S2). The high coefficients of determination (R^2^), in the range from 98.2 to 99.8%, confirmed that the model had a very low variability and that only less than 2% of the total variation was not explained by the model itself (Additional file 2: Table S2). The low p values of some coefficients (*p* < 0.05) indicated their significance and only the stirring in case of *Sso*Pox 3 M was considered a term not significant for the process (Additional file 2: Table S2). No significance were noted correlating the stirring and the temperature (*Data not shown*). Once determined the optimal parameter ranges for the thermal precipitation of the two enzymes, the processes of large scale *Sac*Pox and *Sso*Pox 3 M thermal precipitation were set up using the more restrictive conditions (4.8 ± 0.5 g·L^− 1^ as initial total protein concentration, diluting the *Sac*Pox and *Sso*Pox 3 M crude with 3 and 5 volumes of buffer, respectively, and the temperature in the range from 66.0 to 72.0 ± 0.5 °C); in these conditions the recovery of both enzymes in large scale was around 85% and the purification improved of 3 folds (Table 1a-b). Also SDS-PAGE analyses of the thermal precipitated samples showed an increase in the representativity of the enzyme bands between 2.6 and 4.6 folds, compared to the crude extract (Additional file 3: Figure S1a-c).

**Table 1:** Downstream purification process of *Sac*Pox and *Sso*Pox 3M

Purification step	Total protein (mg)	Total Activity (Units)	Specificity (Units/mg)	Enzyme recovery (%)	Purification fold
a					
Crude Extract	29150 ± 1457	12748 ± 637	0.44 ± 0.02	n.a	n.a
Thermo-precipitate	8352 ± 250	10793 ± 324	1.29 ± 0.04	84.67 ± 2.54	3.01 ± 0.09
Retentate 100 kDa	4000 ± 80	751 ± 15	0.19 ± 0.004	5.89 ± 0.12	n.a
Permeate 100 kDa	4349 ± 174	10045 ± 402	2.31 ± 0.10	78.80 ± 3.10	5.37 ± 0.20
Retentate 5 kDa	3000 ± 60	9843 ± 196	3.28 ± 0.06	77.21 ± 1.54	7.63 ± 0.15
Permeate 5 kDa	1289 ± 26	201 ± 4	0.15 ± 0.003	1.50 ± 0.03	0.34 ± 0.007
b					
Crude Extract	43560 ± 1306	58355 ± 1750	1.34 ± 0.04	n.a	n.a
Thermo-precipitate	12273 ± 613	49421 ± 2471	4.06 ± 0.20	84.69 ± 4.23	3.03 ± 0.15
Retentate 100 kDa	7002 ± 175	3754 ± 94	0.53 ± 0.01	6.43 ± 0.16	0.40 ± 0.10
Permeate 100 kDa	5201 ± 104	45661 ± 913	8.80 ± 0.18	78.25 ± 1.56	6.70 ± 0.13
Retentate 5 kDa	2663 ± 53	45133 ± 902	17.00 ± 0.34	77.34 ± 1.54	13.10 ± 0.26
Permeate 5 kDa	2520 ± 25	480 ± 4.8	0.19 ± 0.002	0.80 ± 0.008	0.15 ± 0.0015

Downstream purification process of *Sac*Pox (a) and *Sso*Pox 3 M (b) enzymes from fed-batch fermentation biomasses (600 g_cww_), induced with 10.0 mM galactose: total protein content, total enzyme activity, specificity, enzyme recovery and purification fold at the different steps

### Purification by ultra-filtration or by gel-filtration chromatography

**Fig. 6:**
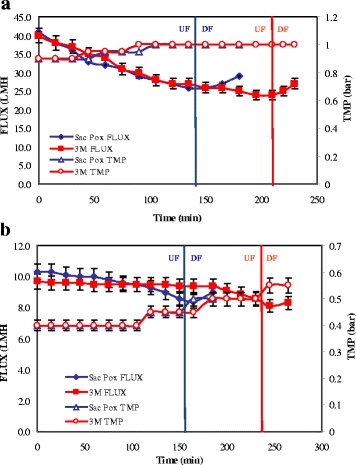
Ultra-filtration and dia-filtration processes on 100 (**a**) and 5 KDa (**b**) membranes of the thermal precipitated samples of *Sac*Pox and *Sso*Pox 3 M

Ultra-filtration processes were set up to recover and to purify the enzymes after the thermal precipitation by using two consecutive steps on 100 and 5 KDa cut-off membranes. The processes on 100 KDa resulted very similar for the samples of the two enzymes in terms of permeate flux decrease, that was around 30% in both processes from 41.0 to 29.0 LMH for *Sac*Pox and from 40.0 to 27.0 LMH for *Sso*Pox 3 M, and in terms of transmembrane pressure (TMP) (Fig. 6) (The *Sso*Pox 3 M process resulted longer due to the major volume to be filtered). The *Sac*Pox and *Sso*Pox 3 M enzymes were collected in the 100 KDa permeate with a 93% of recovery and a purification fold of 5.40 and 6.70, respectively (Table 1a-b). According to the SDS-PAGE analyses, in this step the band representativity for *Sac*Pox and *Sso*Pox 3 M increased correspondently of 4.8 and 5.6 folds compared to the crude extract (Additional file 3: Figure S1 a-c). The permeate volumes were then concentrated and dia-filtrated on 5 KDa cut-off membranes; also in this case the two processes resulted similar, the permeate flux was in the range between 8 and 10 LMH and a TMP between 0.4 and 0.6 bar (Fig. 6). Both enzymes were collected in the retentate volumes with a final recovery of around 98%. The specificity increased up to 3.28 ± 0.20 U·mg^− 1^ and 17.00 ± 0.10 U·mg^− 1^ and the purification fold values resulted to be about1 7.5 and 13.1 for *Sac*Pox and *Sso*Pox 3 M, respectively. (Fig. 6; Table 1a-b). After the process the enzymes resulted active and pure at 75.3 ± 2.0%, according the enzymatic assays, while a pureness of about 81.6% for *Sac*Pox and 77.9% for *Sso*Pox 3 M was determined by SDS-PAGE analyses (Additional file 3: Figure S1a-c). The enzymes were freeze-dried and the lyophilized materials were stored at room temperature: S*ac*Pox resulted 100% active up to 10 months of storage while *Sso*Pox 3 M showed an initial 16% decrease of activity after 8 months of storage. The ultra-filtration process of the thermal precipitated samples was compared with a step of purification on a gel-filtration chromatography system; by loading 15 ml of samples at once, in a single run, the purification folds were around 20.0 ± 1.0 for both enzymes but the recovery was only 45 ± 2.0% (*Data not shown*).

## Discussion

Thermophilic phosphotriesterase-like lactonase enzymes are considered promising and environmental-friendly bioremediation tools for the decontamination of polluted ecosystems from the organophosphate pesticides and good bioscavengers for public and personal safety and protection in case of chemical war attacks [1–3]. They are more stable and resistant than their mesophilic protein counter parts and in the last 10 years diverse engineering strategies have been applied to increase their catalytic activity even of 100 folds. Although that, they are still not commercially available and to be really competitive and industrially attractive they should be produced in high yields, at low cost and at industrial scales [1–3, 13–18]. Thus there is an urgent need of new biotechnological manufacturing processes for their production and purification. So far only one PPL enzyme (*Sso*Pox W263F) has been produced in high yields on large scale [19]. In this paper we aimed to design and extent customize the biotechnological process of production and purification of two different recombinant enzymes*,* the *wild type Sac*Pox and the mutated *SsoPox* 3 M, expressed in *E. coli* [16, 28]. Considering the different substrate specificity and versatility of these two enzymes in degrading OPs and their ability to operate also in harsh conditions and on different surfaces, one of their possible commercial application could be in formulations in which they are present together in a mixture [16, 28]. First of all we optimised the induction conditions by exploring galactose as an alternative inducer to IPTG. Galactose has been already used in high cell density fed-batch fermentations to increase the recombinant protein production up to 10 folds, compared to IPTG, but differences in recombinant protein production could be observed according to the concentrations used [19, 38, 39]. In our shake flask experiments the use of galactose boosted the enzyme expression but in different ways according to the strain and the concentrations of galactose used: 5.0 mM was more effective on the *Sso*Pox 3 M production than on the *Sac*Pox one but the production of both enzymes was greatly boosted, up to 5.2–5.9 folds by using a 10.0 mM concentration. Similarly in the batches of both strains the employment of galactose caused a lower growth inhibition than IPTG and a higher and prolonged enzyme production during the fermentation with a maximum obtained by using a 10.0 mM galactose concentration. But differences were noted between the two strains: the kinetic of *Sac*Pox production was quicker but the *Sso*Pox 3 M final enzyme concentration was the double than the *Sac*Pox one. To further increase the growth, and to reach high cell density values before induction, fed-batch fermentation strategies were coupled with the galactose induction system. This efficient approach resulted to be robust in 2.5-L vessels and scalable in 22.0-L fermentations: the optimized aeration and feeding profiles avoided the formation of acetate at inhibiting levels and allowed to increase the growth and to further boost the production of 5.5 folds for *Sac*Pox and of 11.3 folds for *Sso*Pox 3 M, compared to the batch experiments, up to 2261.0 ± 113.0 U·L^− 1^ and 8673.0 ± 433.6 U·L^− 1^, values 25 and 27 times higher than the ones reported so far in literature, respectively [16, 28]. In the experimental work we also set up a downstream purification process starting with the extraction of the proteins from the cells and taking advantage of their thermal stability. The process should have been more efficient and cost effective than the small scale previous reported ones that included three thermal treatments at different temperatures and at least two chromatographic separation steps to purify the enzymes [16, 28]. The response surface methodology is a good statistical method to optimize the conditions in purification processes of macromolecules [45, 46] and in this study it allowed us to determine the best conditions to obtain a very high recovery (> 95%) of the sulfolobal enzymes from the crude extracts, with a purification fold of 3.0, by using only one thermal precipitation step at 70 °C, thus reducing the costs of the whole purification process of one third in this step. In the optimization study it resulted clear that the initial total protein concentration strictly influenced the recovery yield of both enzymes, as well as the necessity to perform the process in a correct, strict temperature range, while the stirring conditions resulted less critical for the process. The following two-step membrane process allowed to further increase the purification fold up to 7 and 11 times for *Sac*Pox and *Sso*Pox 3 M, respectively, in short times, and to reach a sound final enzyme recovery and pureness, good enough to be used as a decontaminating tools and bioscavenger. The use of membranes is easy to be scaled-up, less time consuming, more economic and adapt than chromatography to purify high volumes of enzyme solutions from 20000–50000-L industrial fermentation vessel implants. The size-exclusion chromatography methods could purify only few milliliter volumes of sample at once, their fluxes are up to five times lower than in ultrafiltration (e.g. 2 ml·min^− 1^), they could take a time up to 25 folds higher than ultrafiltration and thus they obviously are not scalable for pilot and manufacturing plant-size capacity. We calculated that the amount of *Sac*Pox or *Sso*Pox 3 M enzymes obtained in only one manufacturing process, from a 22.0-L fed-batch fermentation and the following purification, would be sufficient to decontaminate from paraoxon and methyl paraoxon a surface 10000 fold wider and to neutralize a quantity of nerve gas cyclosarin 9500 fold higher than the amounts that could be degraded so far with small scale preparation of these enzymes [16]. Besides the enzymes obtained in our process resulted stable for at least 8–10 months as lyophilized material, and that could facilitate their commercialization.

## Conclusions

In conclusion a new, reliable biotechnological manufacturing process of the two recombinant enzymes *Sac*Pox and *Sso*Pox 3 M has been designed and set-up up to the pre-industrial scale by coupling a new galactose-based induction approach with high cell density fed-batch fermentation strategies to produce them, and by coupling one step of a thermal treatment with an ultra-filtration membrane based process to efficiently purify them.

## Additional files

Additional file 1: **Table S1.** Box-Behnken experimental design and results of the thermal precipitation step of *Sac*Pox (a) and *Sso*Pox 3M (b) enzymes by using three independent variables (A-total protein concentration, B-stirring and C-temperature) at three different levels (-1;0;1) (corresponding to 0.48; 4.8; 48.0 g·L-1 for the total protein concentration, 500; 900 and 1200 rpm for the stirring; 60; 70; 80 °C for the temperature, respectively). (DOCX 49 kb)

Additional file 2: **Table S2.** Analysis of the response surface model employed for the optimization of the thermal precipitation step of *Sac*Pox (a) and *Sso*Pox 3M (b) enzymes. (DOCX 61 kb)

Additional file 3: **Figure S1. **SDS-PAGE analyses of the different steps of the downstream purification processes of the *Sac*Pox (a) and *Sso*Pox 3M (b) enzymes from biomasses of fed-batch fermentation induced with 10.0 mM galactose: lane 1-ladder, lane 2-crude extract, lane 3-thermal precipitated sample, lane 4-retentate on 100 kDa, lane 5-permeate on 100 kDa, lane 6-retentate on 5 kDa; lane 7-permeate on 5 kDa; lane 8-immunoblotting of retentate on 5 kDa with specific antibodies. Percentage of representativity of the *Sac*Pox and *Sso*Pox 3M enzyme bands in the different purification steps (c). (PDF 309 kb)

## Abbreviations

AChE: Acetylcholinesterase
BSA: Bovine serum albumin
CWNAs: Chemical warfare nerve agents
DCU: Digital control unit
HPAE-PAD: High performance anion exchange chromatography
HPLC: High performance liquid chromatography
HRP: Horseradish peroxidase
IPTG: Isopropil-β-D-1-tiogalattopiranoside
LB: Luria broth
OPs: Organophosphates
PLL: Thermophilic phosphotriesterase-like lactonase
PVDF: Polyvinylidene difluoride
*Sac*Pox: Sulfolobus acidocaldarius paraoxonase
*Sso*Pox: Sulfolobus solfataricus paraoxonase
TFF: Tangential flow filtration system
TMP: Trans membrane pressure
