# Supplementary material for: High yield production and purification of two recombinant thermostable phosphotriesterase-like lactonases from Sulfolobus acidocaldarius and Sulfolobus solfataricus useful as bioremediation tools and bioscavengers
Source: BMC Biotechnol. 2018 Mar 20;18:18. doi: 10.1186/s12896-018-0427-0 (PMC5861644; doi:10.1186/s12896-018-0427-0)
Supplement: Supplementary file 1 — Table S1. Box-Behnken experimental design and results of the thermal precipitation step of SacPox (a) and SsoPox 3M (b) enzymes by using three independent variables (A-total protein concentration, B-stirring and C-temperature) at three different levels (-1;0;1) (corresponding to 0.48; 4.8; 48.0 g·L-1 for the total protein concentration, 500; 900 and 1200 rpm for the stirring; 60; 70; 80 °C for the temperature, respectively). (DOCX 49 kb) [file 12896_2018_427_MOESM1_ESM.docx]

**Table S1.**

Box-Behnken experimental design and results of the thermal precipitation step of *Sac*Pox (a) and *Sso*Pox 3M (b) enzymes by using three independent variables (A-total protein concentration, B-stirring and C-temperature) at three different levels (-1;0;1) (corresponding to 0.48; 4.8; 48.0 g·L^-1^ for the total protein concentration, 500; 900 and 1200 rpm for the stirring; 60; 70; 80 °C for the temperature, respectively).
