# Supplementary material for: High yield production and purification of two recombinant thermostable phosphotriesterase-like lactonases from Sulfolobus acidocaldarius and Sulfolobus solfataricus useful as bioremediation tools and bioscavengers
Source: BMC Biotechnol. 2018 Mar 20;18:18. doi: 10.1186/s12896-018-0427-0 (PMC5861644; doi:10.1186/s12896-018-0427-0)
Supplement: Supplementary file 2 — Table S2. Analysis of the response surface model employed for the optimization of the thermal precipitation step of SacPox (a) and SsoPox 3M (b) enzymes. (DOCX 61 kb) [file 12896_2018_427_MOESM2_ESM.docx]

**Table S2.**

Analysis of the response surface model employed for the optimization of the thermal precipitation step of *Sac*Pox (a) and *Sso*Pox 3M (b) enzymes.
