# Supplementary material for: High yield production and purification of two recombinant thermostable phosphotriesterase-like lactonases from Sulfolobus acidocaldarius and Sulfolobus solfataricus useful as bioremediation tools and bioscavengers
Source: BMC Biotechnol. 2018 Mar 20;18:18. doi: 10.1186/s12896-018-0427-0 (PMC5861644; doi:10.1186/s12896-018-0427-0)
Supplement: Supplementary file 3 — Figure S1. SDS-PAGE analyses of the different steps of the downstream purification processes of the SacPox (a) and SsoPox 3M (b) enzymes from biomasses of fed-batch fermentation induced with 10.0 mM galactose: lane 1-ladder, lane 2-crude extract, lane 3-thermal precipitated sample, lane 4-retentate on 100 kDa, lane 5-permeate on 100 kDa, lane 6-retentate on 5 kDa; lane 7-permeate on 5 kDa; lane 8-immunoblotting of retentate on 5 kDa with specific antibodies. Percentage of representativity of the SacPox and SsoPox 3M enzyme bands in the different purification steps (c). (PDF 309 kb) [file 12896_2018_427_MOESM3_ESM.pdf]

a

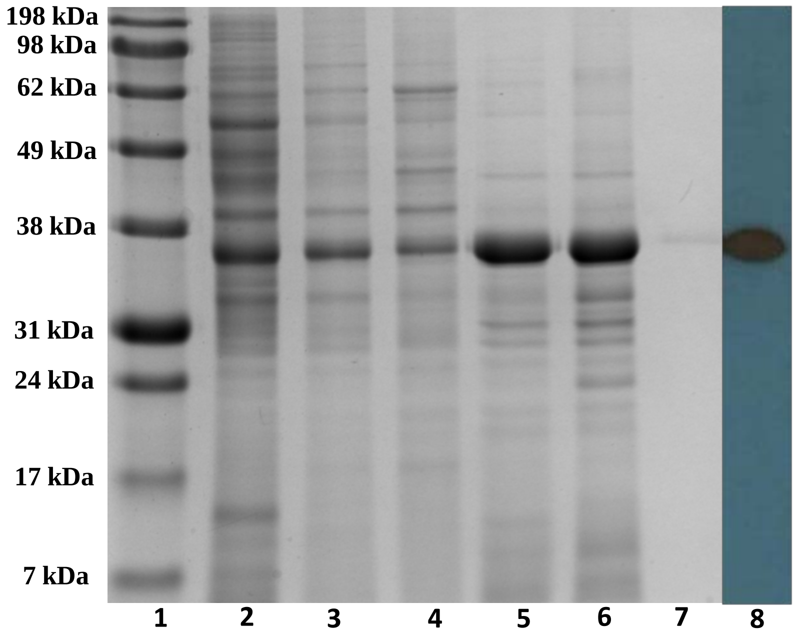

b

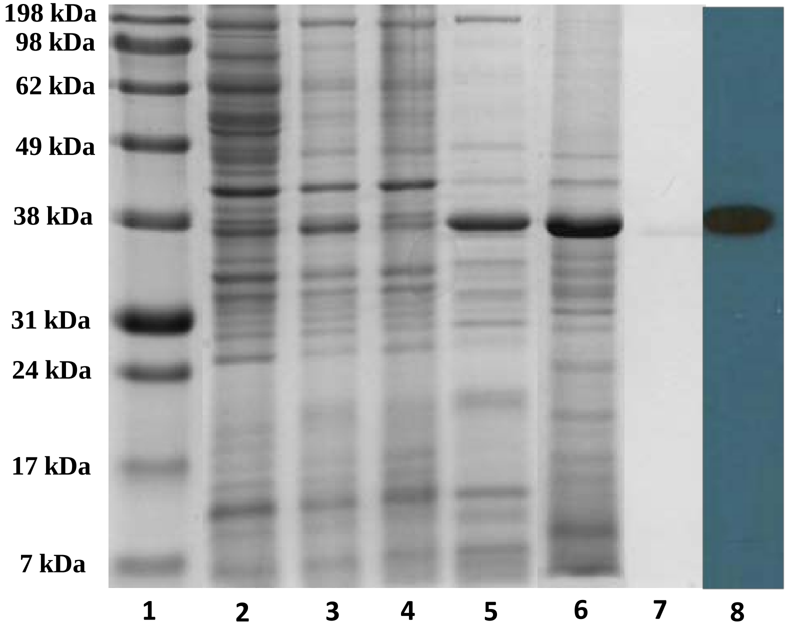

c

Purification Step	Representativity *Sac*Pox (%)	Representativity *Sso*Pox 3M (%)
CrudeExtract	13.6	8.6
Thermal precipitate	35.8	39.7
Retentate 100 kDa	10.3	7.8
Permeate 100 kDa	65.1	48.6
Retentate 5 kDa	81.6	77.9
